# Evaluating the Therapeutic Dose Distribution of Intensity-Modulated Radiation Therapy for Head and Neck with Cone-Beam Computed Tomography Image: A Methodological Study

**DOI:** 10.1155/2014/326532

**Published:** 2014-08-17

**Authors:** Guang-shun Zhang, Shao-min Huang, Cui Chen, Sen-kui Xu, Dan-dan Zhang, Xiao-wu Deng

**Affiliations:** Department of Radiation Oncology, Sun Yat-Sen University Cancer Center, State Key Laboratory of Oncology in Southern China, Collaborative Innovation Center for Cancer Medicine, 651 Dong Feng Road East, Guangzhou 510060, China

## Abstract

An approximate correction method for the CT value-electron density curve of CBCT was established, through comparison and fitting with FBCT images, and applied to evaluate the therapeutic dose of IMRT. The precision of using CBCT for plan calculation was validated by comparing the dose distribution between CBCT- and FBCT-based IMRT plans. Also setup deviations were simulated to evaluate the ability of the CBCT-based calculation for detecting the dose errors caused by positioning deviation. The gamma comparison between CBCT- and FBCT-based dose computations showed that the pass rates of (2%, 2 mm) criteria were better than 97.60 ± 0.83% and 97.74 ± 2.08% in the phantom and 10 NPC cases. When setup deviation was introduced into CBCT-based dose calculation, the gamma pass rate significantly decreased while the volumetric doses of the targets and some normal organs exhibited different changes compared to the original plan. Our results validated the above CT value-electron density correction which reduced the difference between CBCT- and FBCT-based IMRT plan calculation for NPC to less than 2%. Online CBCT-based dose calculation can be used to reflect and evaluate the dose distribution discrepancy caused by setup deviation and structure changes during the treatment, ensuring more effective quality control of IMRT treatment.

## 1. Introduction

Deviation in dose distribution may exist in the delivery of a precisely designed intensity-modulated radiation therapy (IMRT) plan with several causes, including the mechanical inaccuracy of the therapeutic equipment, errors in the output dose characteristics, setup deviation, and anatomical changes of patients during the treatment process. The traditional phantom measurement and validation method can be used to largely detect and avoid dose errors caused by the therapeutic equipment factors; however, to effectively monitor and control the dose errors related to the patient's positioning and structure changes during the treatment remain a challenge. In recent years, with the development in radiotherapy equipment, cone-beam computer tomography (CBCT) technology with online X-ray imaging device equipped on accelerators can reflect information on setup deviation and organ changes of the patient during treatment. However, due to the limitations of the CBCT reconstructing technique, the scattering artifacts in the images are serious, resulting in relatively large deviations in CT values of CBCT from that of fan beam computer tomography (FBCT) for the material with same electron density. Therefore, currently, CBCT images are mainly applied for image-guided setup correction in the clinic and non-scattering-corrected CBCT images cannot be directly used in dose calculation [1, 2]. Relevant studies have indicated that CT value correction based on the corelationship of CBCT and FBCT could bring a closer dose distribution results calculated using the two kinds of CT image [3]. This study used an anatomical equivalent head phantom to establish an approximate model of CT value correlation between CBCT and FBCT images. This method could be used to correct the CT value-electron density curve of CBCT. In addition, validation and evaluation of the calculation errors in the dose distribution of IMRT planning were performed using CBCT images of simulation phantoms and actual patient images, respectively.

## 2. Materials and Methods

### 2.1. Anatomical Head Phantom Used for Simulations

A Chinese human head phantom (subsequently identified as “the head phantom”) was used to simulate irradiation performance, which was developed and produced by the Institute of Ergonomics and Medical Equipment of Sichuan University. The phantom referenced the geometrical size of Chinese adult heads and used tissue equivalent material with similar radiation absorption and scattering to human tissues. Simulated structures such as bone and cavities were installed inside.

### 2.2. CT Image Acquisition

CT scanning images of the head phantom and 10 clinical patients were acquired for analysis in this study. All patients were nasopharyngeal carcinoma (NPC) cases receiving IMRT treatment in our department during the period between August and November of 2012. During the collection of images, the head phantom and patients were kept in the supine position in accordance with the requirements of head and neck IMRT treatment. The FBCT and CBCT images were acquired using a CT simulator (Brilliance Big Bore, Philips Healthcare) and an accelerator equipped with an X-ray imaging system (Synergy XVI, Elekta AB), respectively. For FBCT scanning, the tube voltage was set to 140 kV; the FOV was 500 mm; and the reconstruction thickness was 3 mm. For CBCT imaging, the tube voltage was set to 100 kV; the gantry angle was 260–100 degrees; an S20 collimator without a filter was used; and the reconstruction thickness was also 3 mm. The acquired images were transmitted to the treatment planning system (TPS) for documentation and storage.

### 2.3. Correction of the CT Value-Electron Density Relationship of CBCT Image

The above acquired FBCT and CBCT images of the head phantom were processed in the TPS (Eclipse V10.0, Varian Medical) for automatic image registration. Through the surface marker evaluation and manual adjustment of the registration effect of these two sets of images, the precise alignment of the geometrical structure of these two sets of images was ensured as much as possible. Twenty corresponding slices on the FBCT/CBCT images after registration were randomly selected, and five pixel points were randomly selected from each slice image. As a result, the CT values at the 100 corresponding points in the FBCT and CBCT images (FBCT# and CBCT#) were obtained. Subsequently, the CT values at the corresponding points were plotted using FBCT# as the horizontal axis and CBCT# as the vertical axis, and the relationship of these two CT data sets was then fitted to obtain an approximate function between the two. Based on the internal FBCT#-electron density curve in TPS and the above fitting function, the corrected CBCT#-electron density approximate curve was obtained after conversion. This corrected CBCT value-electron density curve was then introduced into the TPS for CBCT-based dose computation later on.

### 2.4. The Validation of the Dose Precision Calculated Using the Approximately Corrected CBCT Value-Electron Density Curve

#### 2.4.1. Validation of the CBCT-Based Dose Calculation with the Head Phantom

The FBCT and CBCT images, as well as the corresponding CT value-electron density curves, were used to calculate the same IMRT plan, and comparison was performed for the dose distribution on transversal, sagittal, and coronal planes at the isocentric position, including isodose lines on the three planes and the gamma pass rates between the two calculated results.

10 IMRT plans of head and neck patient were tested to compare the dose calculation on FBCT and CBCT, using hybrid plan on the head phantom images of FBCT and CBCT, respectively; the gamma pass rates evaluation for the dose distribution comparison was performed using a commercial software (OmniPro ImRT V1.7b, IBA Dosimetry).

#### 2.4.2. Validation of IMRT Dose Calculation with CBCT Images of NPC Patients

The clinical IMRT treatment plans (FBCT-based plans) of 10 NPC patients were randomly selected and the CBCT images acquired at the first treatment of each patient were introduced into TPS for dose calculation (CBCT-based recalculation). The dose distribution and gamma pass rates on the three planes at the isocentric position between the CBCT-based recalculation and FBCT-based plan were compared.

#### 2.4.3. Evaluation on the Sensitivity of CBCT-Based Dose Checking to the Patient Setup Deviation

CBCT image can be used to assess the effect on delivered dose distribution of IMRT treatment positioning, since it reflects the positioning deviation of patient. The IMRT treatment plan and the CBCT images of the first treatment setup of one NPC patient were randomly selected. Deviations of 1 mm–3 mm were introduced into the CBCT images at the left to right (*X*), superior to inferior (*Y*), and anterior to posterior (*Z*) directions to simulate patient setup deviation. The dose calculation was conducted on the CBCT image with simulated deviation and compared to the original plan using gamma pass rate evaluation. The contour of each target and the involved organs of the treatment plan were mapped to the CBCT images from original plan to evaluate the changes in the dose volume histogram (DVH) in each treatment target and involved organs.

## 3. Results

### 3.1. The Approximate Correction of the CBCT Value-Electron Density Relationship

**Figure 1:**
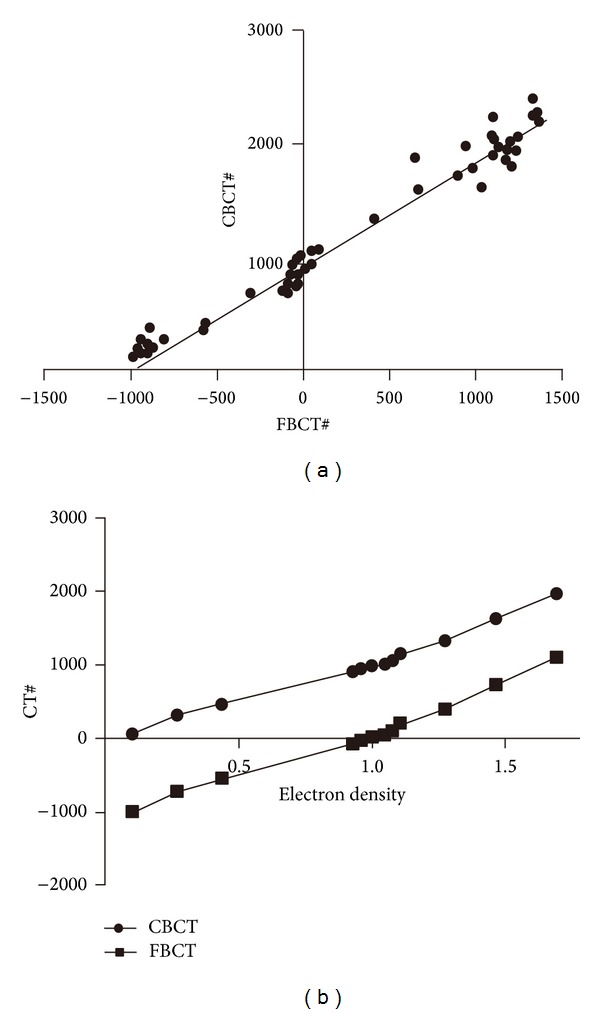
The relationship between FBCT# and CBCT# (a) and the CT value-electron density curve of FBCT and CBCT (b).

The CT values at the randomly selected 100 corresponding points in the FBCT and CBCT images displayed a relatively close linear relationship (Figure 1(a)). The linear regression fitting between the two derived the following approximate function:

(1) $$\begin{array}{c} \text{CBCT}\#=951.8+0.908\times \text{FBCT}\#,\;\left(\text{Correlation}=0.985\right). \end{array}$$

The fitting formula and fitting parameters all had statistical significance (*P* < 0.01).

Using the above fitting formula and based on the CT value-electron density curve of FBCT, the FBCT# corresponding to the electron density of each tissue was converted into CBCT#. The CT value-electron density correction curve of CBCT was then obtained (Figure 1(b)).

### 3.2. Comparison between CBCT- and FBCT-Based Dose Calculations on the Head Phantom

In the head phantom hybrid IMRT plan test, the recalculated dose distribution using CBCT images were compared to the FBCT-based result (Table 1). The average gamma pass rates of the dose calculation on the three isocentric planes were 99.86 ± 0.05%, 99.84 ± 0.21%, and 99.50 ± 0.27% (3%—3 mm criteria) and 98.68 ± 0.52%, 97.60 ± 0.83%, and 98.81 ± 0.42% (2%—2 mm criteria), respectively. The results of the two groups had very high consistency.

### 3.3. The Validation of CBCT-Based Dose Calculation with Clinical Patient's Images

Using the above described approximate correction of the CBCT value-electron density relationship, the CBCT images of 10 clinical NPC patients acquired at the first treatment setup were used to recalculate the dose distribution and the results were then compared with the original FBCT-based treatment plan (Table 2). The average gamma (3%, 3 mm) pass rates for the dose distribution at the three isocentric planes between the CBCT- and FBCT-based plans were 99.89 ± 0.18%, 99.95 ± 0.08%, and 99.86 ± 0.28%. Even when the (2%, 2 mm) criteria were used, the average pass rates remained higher than 97%.

**Figure 2:**
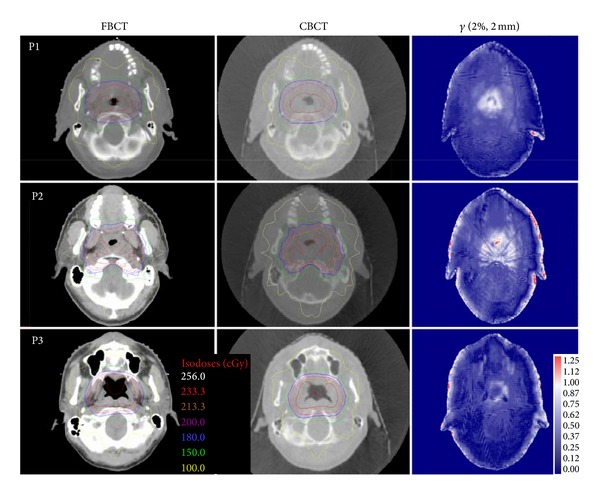
Comparison of dose distribution and gamma values on the transversal plane at the isocenter position between the two plans based on FBCT and CBCT images for three patients (P1–P3). On the right (the third column) are the gamma comparison results (2%, 2 mm) of these two plans. The red areas denote pixels with gamma values greater than 1.

Figure 2 compared the dose distribution in CBCT- and FBCT-based plans on the transversal plane of three randomly selected patients. The figure shows that the differences in the shape and range of isodose lines between the CBCT-based and FBCT-based plans were extremely slight. The points with a larger deviation were primarily concentrated at the edges of the images and in the vicinity of the air chambers of the nasopharyngeal cavity, with dose differences less than 2%.

### 3.4. The Detection Ability of CBCT-Based Dose Checking regarding the Dose Changes Caused by Positioning Setup Deviation

**Figure 3:**
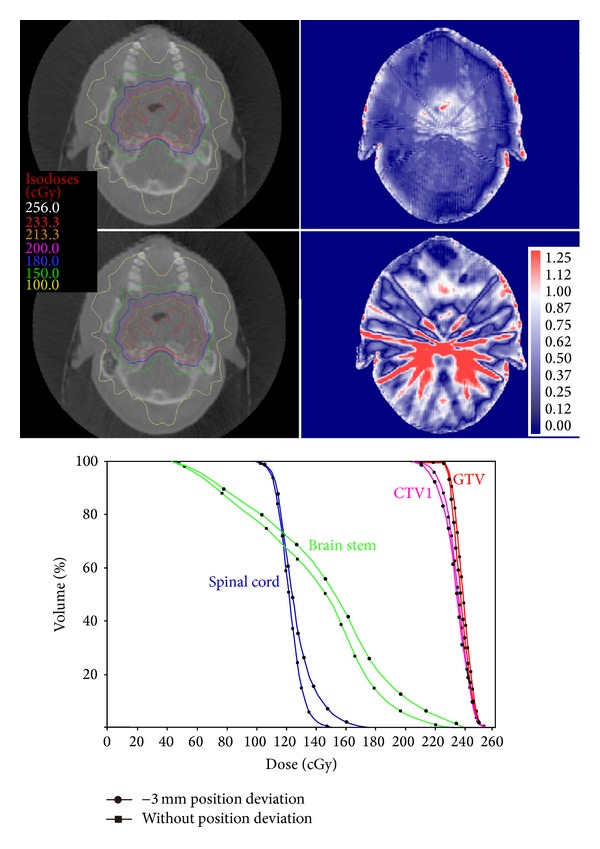
The isodose line (left), gamma (2%, 2 mm) comparison (right) on the transversal plane, and DVH changes for the simulation condition with a −3 mm positioning deviation along the A-P direction. The upper row is the result of correct positioning and the second row is the result with positioning deviation.

One case of NPC IMRT plan and the CBCT images with the first treatment setup were randomly selected and the above-mentioned method was used to simulate setup deviations of 1 mm, 2 mm, and 3 mm in three directions (superior-inferior S-I, anterior-posterior A-P, and left-right L-R). The comparison between the CBCT-based dose calculation with the introduction of the setup deviation and the original treatment plan showed that when the setup deviation was in the range of 1 mm-2 mm, the change in the gamma (3%, 3 mm) pass rate was small, while the change in the gamma (2%, 2 mm) pass rate was significant. When the setup deviation reached ±3 mm, the gamma (3%, 3 mm) pass rate was still over 95% but the gamma (2%, 2 mm) pass rate was reduced to approximately 75% (Table 3). When the deviation of patient positioning at the anterior-posterior direction reached 3 mm, the CBCT-based dose recalculation showed that the DVH of the target and certain normal organs changed significantly. The dose received by the target volume was reduced (3 mm setup deviation towards the anterior direction) or the maximum dose of the spinal cord significantly increased (3 mm setup deviation towards the posterior direction), as shown in Figure 3.

## 4. Discussion

The accuracy of the dose distribution for IMRT is affected not only by the planning design and the uncertainties of the therapeutic equipment but also by the patient position deviation during the treatment process. In addition, the accuracy is also influenced by changes in the shape and location of anatomical structures in the patient body, such as the changes caused by tumor shrinkage or weight loss. These changes cannot be evaluated using the traditional phantom measurement methods. The online CBCT images can more effectively reflect changes in the body position and anatomical structures of patients. However, because of having more serious scattering artifacts the CBCT images do not provide correct Hounsfield units (HU) and cannot be directly used for the dose calculation [4].

By applying image registration, this study proposed an approximate correction method based on the fitting between the CT values of FBCT and CBCT images. The corrected CT values of the CBCT images were very close to that of the corresponding FBCT images, with almost all deviations less than 200 HU from the FBCT value. To select a suitable number of matching points for controlling the uncertainty of CBCT# approximation, we compared the fitting results of four different sampling groups: fitting from 60, 80, 100, and 120 matching points. As shown in Table 4, the fitting parameters tended to be consistent when the number of match points was larger than 80. In this study a number of 100 matching points were selected. The fitting correlation coefficient was 0.985 with a standard deviation of 111.9 HU in the difference between the approximated CBCT value and corresponding FBCT value, and a mean difference of zero was obtained. That meant that about 70% of the differences between the corrected CBCT# and the FBCT# were within ±111.9 HU and 95% within ±223.8 HU.

Based on the CBCT#-electron density curve obtained in this study, the largest gradient coefficient of the changes between the electron density and CBCT# was 0.088/100 HU; that is, the deviation of the relative electron density was only 0.088, while the deviation of the CT value was 100 HU. Moreover, in a certain relatively flat region of the curve, the deviation of the electron density was even smaller. Previous study [5] reported that for single-field irradiation, when the deviation of the CT value in dense bone was 100 HU and in soft tissue organs was around 30 HU, the resulted deviation in the dose calculation was smaller than 1%. For IMRT planning, the effect of deviation in CT value on dose calculation was even slighter. In this study, we conducted CBCT-based dose calculation for IMRT plans on the head phantom and observed only slight differences in the results compared with the FBCT-based calculation. The comparison of these two calculation methods showed that the average gamma pass rate for 10 hybrid phantom plans at the 3%—3 mm and 2%—2 mm criteria was better than 99.48% and 97.60%, respectively. van Zijtveld et al. [3] reported a method to correct the CT value of CBCT by mapping the HU number from FBCT to CBCT images. In their study, the gamma (2%, 2 mm) pass rate of the comparison between the CBCT- and FBCT-based IMRT plan calculation for 5 cases was 92%–95%, which was worse than our results. Similar to our study, their results also showed that most differences were located on the body outline area. Rong et al. [6] performed a study using a CIRS phantom to obtain the site specific CBCT value and the CBCT#-electron density curve. Their results concluded the dose accuracy to approximately 2% on the head RANDO phantom, which was similar to this study. Different from Rong's report, in this study, we also performed the dose calculation based on the CBCT images of actual clinical patients acquired during the first treatment fraction. Using the above-mentioned correction method, we obtained results consistent with those of the FBCT-based calculation. The average gamma (3%, 3 mm) pass rate and the gamma (2%, 2 mm) pass rate were better than 99.86% and 97.74%, respectively. Boggula et al. [7] segmented different homogeneous structures in the CBCT images and assigned reasonable averaged HU to them which were derived from the Planning CT and obtained 97% and 96% gamma (3%, 3 mm) pass rates in CBCT-based dose calculation for pelvic phantom and prostate patient images, respectively, compared to FBCT-based calculation. Compared to the above literatures, our method to approximately correct the CBCT#-electron density is easier to apply and gives a better accuracy in dose calculation.

Certain studies reported the use of standardized CT density phantoms to correct the CT values of CBCT images for dose calculation [8–10]. Because the distribution and size of the scattering artifacts of CBCT vary with the body shape and position of the phantom or patients, it is difficult to determine the discrepancy in CBCT-based calculation using homogeneous phantoms. A head phantom simulating the Chinese adult, which was manufactured in Sichuan, China, was used as the material for the correction experiments in our study. This phantom processes a structure highly similar to the head tissues of the majority of Chinese patients, thus minimizing the effects caused by differences in anatomical structure, location, and volume on the accuracy of the CT value correction during CBCT-based dose calculation.

Our results showed that although this CT value-electron density curve correction method could improve the CBCT-based dose calculation to be similar to the FBCT-based computation, there was still some dose deviation on the body outline region or the surface of the cavities. A possible reason was that, compared with FBCT images, the image boundary between tissues and air in CBCT images was not clear and the scattering condition in the region with blurred boundaries was relatively complex. As reported previously, this factor could result in an even larger deviation in the dose calculation using pulmonary CBCT images [11]. Another reason was that the marginal dose deviation caused by setup deviation might be relatively larger.

By introducing a simulation of setup deviation, this study explored the sensitivity of an online CBCT-based dose validation method for the evaluation of therapeutic dose errors. The results showed that although the gamma pass rate at the (3%, 3 mm) criteria did not effectively reflect a setup deviation of less than 3 mm, the gamma pass rate at the (2%, 2 mm) criteria showed higher effectiveness in detecting a dose distribution error caused by a setup deviation over 2 mm. More importantly, the proposed method was able to not only detect the gamma pass rate of dose distribution but also provide statistical information on the volume dose and DVH during actual treatment of patients. In Wang's study of using CBCT image to assess the delivery dose deviation in the partial breast irradiation, 47.4% of the treatment fractions reduced the dose coverage according to the posttreatment CBCT-based dose evaluation [12]. The results of our study showed that the CBCT-based dose checking could be used as a tool for finding the dose error caused by patient setup and provide a basis or action reference for adaptive replanning.

During the statistical analysis of DVH information in this study, the changes in tumor and normal organs are negligible at the patient's first treatment because the head and neck part was considered as a rigid structure. Through the registration of CBCT and the planned CT images, the organs of interest were directly copied onto CBCT images for the statistical analysis of DVH information. Nevertheless, this method might produce some minor errors. With the progression of treatment process, organ delineation at the mid and late stage of the treatment course should be performed by means of deformable image registration approach and finally confirmed by clinicians [13–15]. In addition, as this study is based on the experiments with the head phantom and validated with patient images of head and neck area, the reported approximation to correct the CBCT value-electron density curve may not be suitable for CBCT-based dose calculation in thoracic or abdominal cases, in which the structure deformation is larger and further study should be conducted.

## 5. Conclusion

We proposed an approximate method for the correction of the CT value-electron density relationship in CBCT images. Based on CBCT images, this method could be used to validate and evaluate dose distribution under the actual patient setup during treatment. This method could effectively reflect the discrepancy in dose distribution caused by setup deviation and structure changes in actual clinical practice. However, because this dose evaluation approach relies on the calculation results of TPS and does not consider the errors in the accelerator machinery and output dose accuracy, the validation of irradiation dose for the treatment should be performed through the combination of the proposed method and other online measurement methods. The combined approach could achieve superior performance in determining the anatomical location of therapeutic dose errors and in assessing the deviation of volumetric dose parameters, thus greatly improving the clinical applicability and effectiveness of quality control during radiotherapy.

## Figures and Tables

**Table 1:** Gamma pass rate of dose comparison between CBCT- and FBCT-based head phantom hybrid plans for 10 head and neck IMRT cases.

Case number	Transversal		Sagittal		Coronal	
	3%—3 mm	2%—2 mm	3%—3 mm	2%—2 mm	3%—3 mm	2%—2 mm
1	99.75%	97.22%	99.46%	97.49%	99.50%	98.53%
2	99.94%	99.27%	99.57%	97.47%	99.56%	98.98%
3	99.86%	98.83%	99.29%	96.93%	99.27%	98.54%
4	99.89%	98.77%	99.38%	97.37%	99.26%	98.57%
5	99.84%	98.77%	99.36%	97.13%	99.31%	98.47%
6	99.89%	98.79%	100.00%	99.94%	100.00%	99.54%
7	99.84%	98.88%	99.44%	97.45%	99.45%	98.70%
8	99.88%	98.96%	99.21%	97.03%	99.18%	98.35%
9	99.85%	98.60%	99.64%	98.00%	99.98%	99.61%
10	99.84%	98.74%	99.44%	97.22%	99.49%	98.76%

Ave. ± Std	99.86 ± 0.05%	98.68 ± 0.52%	99.48 ± 0.21%	97.60 ± 0.83%	99.50 ± 0.27%	98.81 ± 0.42%

Note: gamma pass rate counted all the points with a dose >10% of the prescribed dose.

**Table 2:** Gamma pass rates on the isocentric planes between CBCT- and FBCT-based IMRT plans with 10 patient's images.

Case number	Transversal plane (T)		Sagittal plane (S)		Coronal plane (C)	
	3%—3 mm	2%—2 mm	3%—3 mm	2%—2 mm	3%—3 mm	2%—2 mm
1	99.98%	99.86%	99.99%	99.95%	99.99%	99.91%
2	99.79%	95.35%	99.99%	99.68%	99.87%	95.10%
3	99.37%	95.05%	99.92%	98.54%	99.08%	94.93%
4	99.99%	98.37%	100.00%	98.93%	100.00%	97.88%
5	99.86%	96.41%	100.00%	100.00%	99.96%	98.70%
6	100.00%	99.60%	100.00%	99.99%	100.00%	99.54%
7	99.96%	97.55%	99.83%	98.11%	99.68%	94.69%
8	100.00%	99.61%	100.00%	99.82%	100.00%	99.89%
9	100.00%	99.87%	100.00%	99.83%	100.00%	99.93%
10	99.92%	98.50%	99.78%	97.33%	100.00%	96.81%

Ave. ± Std	99.89 ± 0.18%	98.00 ± 1.76%	99.95 ± 0.08%	99.22 ± 0.90%	99.86 ± 0.28%	97.74 ± 2.08%

**Table 3:** The effect of simulated setup deviation of patient positioning on the gamma pass rate on the transversal plane at the isocenter position.

Setup deviation		Gamma pass rate	
		3%—3 mm	2%—2 mm
No error	0	99.99%	98.00%

Anterior-posterior direction	1 mm	100.00%	95.12%
	2 mm	99.99%	89.23%
	3 mm	95.31%	75.85%
	−1 mm	99.98%	97.06%
	−2 mm	99.99%	94.78%
	−3 mm	97.64%	83.99%

Superior-inferior direction	1 mm	99.75%	96.42%
	2 mm	99.15%	93.92%
	3 mm	97.76%	88.48%
	−1 mm	99.05%	92.94%
	−2 mm	97.22%	86.99%
	−3 mm	95.29%	79.41%

Left-right direction	1 mm	99.99%	97.25%
	2 mm	99.94%	92.00%
	3 mm	96.42%	78.31%
	−1 mm	99.95%	96.19%
	−2 mm	99.99%	94.59%
	−3 mm	98.06%	75.28%

**Table 4:** Fitting results for different sample numbers of matching points.

Fitting parameters	Number of matching points			
	60	80	100	120
*a* (coefficient)	0.922	0.907	0.908	0.907
*b* (constant)	948.2	950.9	951.8	950.8
Diff. mean	−0.3	−0.4	0.0	0.0
Diff. Std	108.2	116.7	111.9	110.2
*R* (correlation)	0.987	0.985	0.985	0.985
